# Exploring interactions between maturity status and playing time with fluctuations in physical fitness and hormonal markers in youth soccer players

**DOI:** 10.1038/s41598-022-08567-5

**Published:** 2022-03-16

**Authors:** Ebrahim Eskandarifard, Hadi Nobari, Mustafa Sogut, Filipe Manuel Clemente, António José Figueiredo

**Affiliations:** 1 Department of Exercise Physiology, Faculty of Sport Sciences, University of Isfahan, 81746-7344 Isfahan, Iran; 2 Department of Exercise Physiology, Faculty of Educational Sciences and Psychology, University of Mohaghegh Ardabili, 56199-11367 Ardabil, Iran; 3 Department of Physiology, Faculty of Sport Sciences, University of Extremadura, 10003 Cáceres, Spain; 4 Sports Scientist, Sepahan Football Club, Isfahan, Iran; 5 Department of Physical Education and Sports, Faculty of Education, Middle East Technical University, 06800 Ankara, Turkey; 6 Escola Superior Desporto e Lazer, Instituto Politécnico de Viana Do Castelo, Rua Escola Industrial e Comercial de Nun’Álvares, 4900-347 Viana do Castelo, Portugal; 7 Instituto de Telecomunicações, Delegação da Covilhã, 1049-001 Lisboa, Portugal; 8 Faculty of Sport Sciences and Physical Education, University of Coimbra, Coimbra, Portugal

**Keywords:** Biochemistry, Physiology

## Abstract

The purpose of this study was to determine the differences in functional capacities and hormonal indices according to biological maturity and seasonal playing time status in young soccer players. Twenty-three male under-15 players (14.6 ± 0.2 years) were divided into two groups, based on their maturity status and seasonal playing time. They were measured for anthropometrics, Yo-Yo intermittent recovery level 1 (YYIR1), seven repeated sprint test (7RST), countermovement jump (CMJ), skeletal age, growth hormone, and insulin-like growth factor-1 (IGF-1) level. Age at peak height velocity (APHV) was determined to estimate the maturity timing. The results revealed that players who advanced in maturation were significantly heavier (p = 0.029) and had higher values in skeletal age (p < 0.001), sitting height (p = 0.005), CMJ (p = 0.038), and IGF-1 (p = 0.013). Players given greater playing time exhibited significantly lower fatigue index (p = 0.012), but higher CMJ (p = 0.003) and IGF-1 (p = 0.001) values. The overall results highlighted that early-maturing players and players with greater playing time obtained higher values in both CMJ and IGF-1. The findings may provide an insight on the coaches’ evaluation of players and on the possible factors that may affect the future playing status of young male soccer players.

## Introduction

Puberty is a time of substantial developmental changes in the size and composition of the body^1^. The onset and progression of puberty are associated with body mass, body mass index, nutritional behavior, and various genetic and environmental factors^2,3^. Thus, there can be considerable variations in duration, tempo, timing of pubertal maturation, and adolescent growth spurt among typically developing children born in the same calendar year^4,5^. Several studies documented maturity related differences in anthropometric, physical fitness characteristics, and sport-specific skills such as passing, dribbling, and shooting of young soccer players within a chronological age group^6–9^.

Scientific evidence revealed that maturity status plays a significant role in the context of talent identification and selection in youth soccer players^5,10–12^. Since maturity status is not taken into account in the physical and technical talent identification testing procedures, early maturing players generally perform better in these tests and thus they are recognized as more talented. According to Malina et al.^11^**,** late-maturing players are systematically excluded in talent development programs. Besides, their under-representation in certain age groups was reported^7,13^. In a previous study, a significant difference in maturity status was noted in favor of the players who progress to a professional level when compared to their remained amateur counterparts^14^. Correspondingly, available data on young soccer players showed that players advanced in maturation were selected and took part in first teams at elite and regional level^9,15,16^. Thus, maturity status, as an important indicator of biological development, should be integrated the selection process of highly trained adolescent soccer players^17^.

The onset and duration of pubertal growth and biological maturation of adolescent is mainly controlled by the growth hormone (GH), insulin-like growth factor-1 (IGF-1), and thyroid^18^. Although it is difficult to establish a cause-effect relationship between hormonal status and sports-specific skills, higher levels of GH and IGF-1 were found in young soccer players compared with their non-active control counterparts during different pubertal stages^19–21^. This difference might be attributed to the adrenal hyperactivity stimulated by exercise^22^. Muscella et al. (2019) observed soccer training-induced changes in hormonal status in young soccer players^20^. The changes in the hormonal profile may also have important role in sporting performance that, for example, Vänttinen et al.^23^ reported the positive association between hormonal status and sport-specific skills in young soccer players. Nonetheless, the timing and the tempo of the growth and maturation may also be sensitive to other factors such as nutritional status and excessive energy expenditure.

Despite the plethora of research on the development of physical fitness characteristics, current pediatric exercise literature provides limited evidence on the maturity-related differences in hormonal status of players in a single age category. Moreover, there is also a paucity of information on the differences in hormonal activities and physical performance variables between players who have more or less seasonal playing time status. Being constantly exposed to higher competition loads may lead a greater physical and functional development for players with more seasonal playing times. Therefore, the purpose of this study was to determine the variations in functional capacities and hormonal indices according to biological maturity status and playing time in young soccer players. It is hypothesized that early-maturing players and players who have greater playing time status will show better performance in physical fitness tests and higher hormonal profile compared to less mature players and players who have lower playing time status. The findings of the study may help to enhance our understanding on future playing status of young players.

## Methods

### Participants

Twenty-three youth soccer players, mean ± standard deviation (SD), age: 14.6 ± 0.2 years; stature: 173.7 ± 6.6 cm; body mass: 61.0 ± 9.6 kg; skeletal age: 15.1 ± 0.9 years; maturity offset: 1.17 ± 0.48 years; and minutes of playing: 1377.1 ± 267.1) participated in this study. They were playing in the Isfahan U15 Provincial and Iranian Regional League. Position and the number of players in each position were: 3 goalkeepers, 10 defenders, 3 central midfielders, 4 wingers, and 3 attackers. The inclusion criteria were: (1) playing soccer for at least three years; (2) participated in all part of this study; (3) did not take any kind of food supplement which influence on growth, and (4) did not participate in any extra training session. The exclusion criteria were: (1) participating in less than 80% of training and games; (2) absence in any examination or tests of this study; (3) late maturity status. In this study there was only two players with late maturity status therefore we eliminated them to avoid analytical errors. To engage in this study, both the players and their parents signed an informed consent form. The study has approved by the University of Isfahan Ethics Committee prior to its start (IR.UI.REC.1399.001), and the Helsinki Declaration was used to follow the recommendations of Human Ethics in Research.

### Experimental approach to the problem

This study was performed as a quasi-experimental and cohort research which was conducted on a cross-sectional basis. Players were divided into two groups in terms of playing time, the first group played less than 50% of the tournament time (PT1) and the second group played more than 50% of the tournament time (PT2) in fact PT1 and PT2 were separated based upon the amount of match-play^24^. According to the maturity, players were categories as normal maturity (ML1) or early maturity (ML2)^25^. When the subtraction of skeletal age and chronological age (SA-CA) was more than + 1, players were classified as early mature, if SA-CA was between − 1 and + 1, players were classified on time. For determining the minutes of playing, all formal and informal games of player spotted. During this study, players weekly participated in one official or non-official match. At the end of this season after three days’ rest, assessments were performed which those performed respectively at four days and in the morning. In this trend that in the first day we did blood sampling, X-Ray and anthropometric measurement were used to identifying maturity status, furthermore physical fitness performed were the countermovement jump (CMJ), the seven repeated sprint test (7RST), and yo-yo intermittent recovery levels 1 (YYIR1), which were performed on the second, third, and fourth days, respectively. All of these tests were performed in the morning under the same weather conditions (21–23 °C temperature and 50% humidity)^26^.

### Measurements

#### Blood sampling

On the first day of evaluation, performed after three days of non-training, players arrived at the laboratory of Al-Zahra Hospital in Isfahan at 8 o'clock, after 12 h of fasting, to collect 10 ml of blood from the brachial vein to measure GH and IGF-1^27^. Blood samples were taken by a specialist, and then centrifuged and kept at – 70 °C until the analysis. The Chemiluminescence method and IMMULITE system were used to evaluate the samples. The output results were Nano grams per milliliter (ng/mL) for IGF-1 and GH.

#### Skeletal age

They went to the EOS imaging department to take a posterior-anterior X-ray of the hand, and wrist of the left hand to determine the skeletal age. This device had 50–85% less radiation than 2D X-Ray^28–30^ and better image quality^31,32^. The skeletal age of participants was assessed with Fels method by an experienced person. The Fels method is the most reliable method for assessing radiography^33^. After assessment the data inserted into the Fels by version 1.0 to calculate the skeletal age and standard error. Intra-observer error associated with skeletal age assessments was lower than 0.18 year.

#### Anthropometric measurements

At the end of the first day of the assessment in the morning^34,35^, anthropometric measurements of standing height, sitting height, and body mass of players were evaluated. To measure standing height, players standing in front of the Stadiometer without shoes and socks. Their heels, buttocks, shoulders, and head were close to the Stadiometer. For sitting height, players sat on a 50 cm chair and their hips, shoulders, and head were as close as possible to the Stadiometer^36–38^. To measure these two height parameters, a German-made Seca model 213 Stadiometer with an accuracy of 0.005 m was used. In the end, weight was measured by a digital scale. The players were wearing only sports shorts. We used the digital scale of Seca brand Model 813 made in Germany with an accuracy of 0.1 kg. The output results were centimeters (cm) for standing height and sitting height. Ultimately, body mass was recorded in kilograms (kg). The technical error of measurement, inter- and intra-observer, were lower than 5% for standing and sitting height and was lower than 3% for the weight.

#### Maturity measurements

We used anthropometrics measurement for estimating age at peak height velocity (APHV) and maturity offset, for estimating this variable we utilize Mirwald et al. equation, where Maturity offset =  − 9.236 + 0.0002708 (leg length × sitting height) − 0.001663 (age × leg length) + 0.007216 (age × sitting height) + 0.02292 (weight by height ratio), R = 0.94, and R^2^ = 0.891) and for leg length = Standing Height (cm)—Sitting height (cm)^39^. Standard error of the equation is 0.592 year. The output results were years for APHV and maturity offset.

#### Physical fitness tests

CMJ test was performed to assess explosive power of lower body^40^. Before the CMJ test, players warmed up for 15 min, including jogging, stretching, and jumping exercises. Then, they were taught the procedure of the test, and performed 2 CMJ as a familiarization test. During the CMJ test, players stood on the electronic pad with their hands fixed, without swing, on his lateral part of the pelvic, and bent at the knee joint, then they jumped up with the maximum power and after the first test, after 5 min' recovery they did second test. The best of the 2 tests was included for analysis^41^. The Newtest Power Timer pad made in Finland was used to assess in this test. The reliability was calculated by using intra-class coefficient (ICC) was high (≥ 0.93)^42^.

For the 7RST, each participant run the curved path, which is 34.5 m, 7 times at maximum speed, with 25 s recovery time between sets. The difference between the worst and the best time was considered as a fatigue index in this test^43^. The warm-up included 15 min of jogging, dynamic stretching, and short speeds. The measuring device in this test is a New Power timer made in Finland, that will be placed at the beginning and the end of the track. The ICC was ≥ 0.94^44^.

To estimate the VO_2max_, the YYIR1 test was used with the following formula: V̇O_2max_ = IR1 distance (m) × 0.0084 + 36.4^45^. In this test, participants run a distance of 40 m in the form of two 20-m back and forth, then walk 10 m back and forth at a distance of 5 m for recovery. Every new level the speed of the test increased 0.5 km/h, any player who fails to reach the desired point before beep twice was removed from the test, and the last level was recorded for him. To perform this test, the players warmed up for 15 min, which included jogging, stretching, and agility exercises^45^, then they placed at the starting point of the test, and performed test. The test–retest reliability was calculated in this assessment and result generally sufficient (≥ 0.90)^46^.

### Statistical analysis

Using descriptive statistics, variables including mean and standard deviation were calculated. After confirming the normality of the test data (Smirnoff Kolmogorov) and homogeneity of variances (Levene's test), the Multivariate Analysis of Variance (MANOVA) test to compare athletes of early and normal maturity in each of the two groups of players who were played more than 50% and less than 50% were applied. On the other hands, Multivariate analyses of covariance (MANCOVA) were used two times to control the effect of “minutes of playing” and “maturation level” this two factor apply as a covariate,, also partial eta-square (ƞp^2^) were examined to show the magnitude of differences (trivial effect < 0.2, small effect ≥ 0.2; medium effect ≥ 0.5; large effect ≥ 0.8 and above). All variables examine at a significance level of 0.05 and we use SPSS 22 for this study.

### Ethical approval

To engage in this study, both the players and their parents signed an informed consent form. The study has approved by the University of Isfahan Ethics Committee prior to its start (IR.UI.REC.1399.001), and the Helsinki Declaration was used to follow the recommendations of Human Ethics in Research.

## Results

Table 1 shows the descriptive characteristics (mean and SD) of maturity and playing time in groups. However, some variable like total season playing time: 377.1 ± 267.1 min; height: 173.7 ± 6.6 cm; VO_2max_: 49.00 ± 2.75 ml.kg^-1^ min^-1^; IGF-1 level: 482.2 ± 63.3 ng/dl, and for the skeletal age 15.14 ± 0.88 years were obtained.

**Table 1:** Descriptive statistics of playing time and maturity groups in inter-variability of under 15 youth soccer players.

Variables	Total	Playing time groups		Maturation level groups	
		PT1	PT2	ML1	ML2
	n = 23	n = 10	n = 13	n = 16	n = 7
Minutes playing (min)	1377.0 ± 267.1	1141.5 ± 120.6	1558.3 ± 195.8	1316.3 ± 204.4	1515.8 ± 353.3
Chorological age (year)	14.6 ± 0.2	14.5 ± 0.2	14.7 ± 0.1	14.5 ± 0.2	14.7 ± 0.1
Skeletal age (year)	15.1 ± 0.8	14.7 ± 0.8	15.4 ± 0.8	14.7 ± 0.6	16.1 ± 0.4
Soccer training (month)	80.9 ± 24.4	69.3 ± 20.7	89.8 ± 23.8	79.9 ± 20.2	83.1 ± 33.9
APHV (year)	13.4 ± 0.4	13.4 ± 0.5	13.4 ± 0.3	13.5 ± 0.3	13.1 ± 0.4
Maturity offset (year)	1.1 ± 0.4	1.0 ± 0.6	1.2 ± 0.3	0.9 ± 0.4	1.5 ± .0.4
Standing height (cm)	173.7 ± 6.6	173.0 ± 6.9	174.2 ± 6.6	172.0 ± 6.5	177.6 ± 5.3
Sitting height (cm)	90.8 ± 2.9	90.4 ± 3.7	91.1 ± 2.3	89.8 ± 2.3	93.1 ± 3.1
Body mass (kg)	60.9 ± 9.5	59.2 ± 9.4	62.2 ± 9.9	58.5 ± 8.2	67.1 ± 10.1
VO_2max_ (ml kg^-1^ min^-1^)	49.0 ± 2.7	48.3 ± 2.0	49.5 ± 3.1	49.1 ± 2.2	48.7 ± 3.8
7RST (s)	6.8 ± 0.2	6.9 ± 0.2	6.8 ± 0.2	6.8 ± 0.2	6.8 ± 0.3
Best of 7RST (s)	6.5 ± 0.2	6.5 ± 0.2	6.5 ± 0.2	6.5 ± 0.2	6.4 ± 0.1
Worse of 7RST (s)	7.1 ± 0.2	7.2 ± 0.1	7.0 ± 0.2	7.1 ± 0.2	7.0 ± 0.3
Fatigue index (s)	0.5 ± 0.2	0.6 ± 0.2	0.4 ± 0.1	0.5 ± 0.2	0.5 ± 0.1
CMJ (cm)	41.3 ± 4.0	38.9 ± 3.2	43.2 ± 3.7	42.0 ± 3.9	39.8 ± 4.3
GH level (ng/dl)	0.7 ± 0.5	0.6 ± 0.5	0.8 ± 0.6	0.7 ± 0.6	0.7 ± 0.4
IGF-1 level (ng/dl)	482.1 ± 63.2	449.7 ± 57.5	507.1 ± 57.4	492.6 ± 53.3	458.1 ± 81.2

*PT1* group of player who play less than 50 percent of total season, *PT2* group of player who play more than 50 percent of total season, *ML1* the group of player who were on normal maturation, *ML2* the group of player who were early maturation, *APHV* age at peak height velocity, *VO*_*2max*_ maximal oxygen uptake, *7RST* 7 repeated sprint test, *CMJ* counter movement jump, *GH* growth hormone, *IGF-1* insulin-like growth factor-1.

Results of MANOVA, in Table 2 showed no significant between all groups in that skeletal age, football training, sitting height, VO_2max_, 7RST, best of 7RST, worse of 7RST, and GH. In contrast there were significant difference between PT1 and PT2 in the variables of minutes of playing (F = 34.623, p < 0.01, ƞp^2^ = 0.646), fatigue index (F = 7.737, p < 0.05, ƞp^2^ = 0.289), CMJ (F = 11.710, p < 0.01, ƞp^2^ = 0.381) and IGF-1 level (F = 14.559, p < 0.01, ƞp^2^ = 0.434). In the maturation group there were significant differences between ML1 and ML2 in the variables of skeletal age (F = 30.266, p < 0.01, ƞp^2^ = 0.614), APHV (F = 8.169, p < 0.01, ƞp^2^ = 0.301), maturity offset (F = 11.832, p < 0.05, ƞp^2^ = 0.384), CMJ (F = 4.971, p < 0.01, ƞp^2^ = 0.207) and IGF-1 level (F = 7.453, p < 0.05, ƞp^2^ = 0.282) were related to the difference in means between the interaction of groups of playing time and their maturation I mean “Playing time × Maturation level" which was the only IGF-1 that was significantly (F = 4.926, p < 0.05, ƞp^2^ = 0.206) had difference. The results are in Table 2.

**Table 2:** Results of MANOVA to test the effect of playing time and maturity groups in inter-variability of under 15 youth football players.

Variables	Effects of playing time			Effects of maturation level			Effects of playing × maturation		
	F	P	ƞp^2^	F	P	ƞp^2^	F	P	ƞp^2^
Minutes playing (min)	34.6	< 0.001*	0.6	1.0	0.321	0.1	2.3	0.146	0.1
Chorological age (year)	2.3	0.146	0.1	0.1	0.338	0.1	0.1	0.731	< 0.001
Skeletal age (year)	0.8	0.368	< 0.001	30.3	< 0.001*	0.6	2.1	0.165	0.1
Soccer training (month)	3.1	0.096	0.1	< 0.001	0.940	< 0.001	0.0	0.923	< 0.001
APHV (year)	0.1	0.331	0.1	8.2	0.010*	0.3	2.0	0.171	0.1
Maturity offset (year)	0.0	0.888	< 0.001	11.8	0.003*	0.4	2.7	0.119	0.1
Standing height (cm)	0.1	0.806	< 0.001	3.9	0.064	0.2	0.4	0.552	< 0.001
Sitting height (cm)	0.4	0.539	< 0.001	10.0	0.005*	0.3	2.2	0.159	0.1
Body mass (kg)	0.1	0.790	< 0.001	5.6	0.029*	0.2	1.5	0.230	0.1
VO_2max_ (ml kg^-1^ min^-1^)	0.4	0.529	< 0.001	0.1	0.770	< 0.001	0.3	0.576	< 0.001
7RST (s)	0.2	0.625	< 0.001	0.3	0.575	< 0.001	< 0.001	0.874	< 0.001
Best of 7RST (s)	0.2	0.625	< 0.001	1.5	0.241	0.1	0.1	0.753	< 0.001
Worse of 7RST (s)	2.2	0.150	0.1	0.4	0.547	< 0.001	0.0	0.897	< 0.001
Fatigue index (s)	7.7	0.012*	0.3	0.1	0.769	< 0.001	0.1	0.729	< 0.001
CMJ (cm)	11.7	0.003*	0.4	4.1	0.038*	0.2	0.6	0.439	< 0.001
GH level (ng/dl)	0.5	0.498	< 0.001	0.2	0.691	< 0.001	< 0.001	0.971	< 0.001
IGF-1 level (ng/dl)	14.6	0.001*	0.4	7.5	0.013*	0.3	4.9	0.039*	0.2

*APHV* age at peak height velocity, *VO*_*2max*_ maximal oxygen uptake, *7RST* 7 repeated sprint test, *CMJ* counter movement jump, *GH* growth hormone, *IGF-1* insulin-like growth factor-1.

*Significant difference at (p ≤ 0.05).

Finally, a MANCOVA was perform to test the effect of maturity groups in the inter-variability of U15 youth football players, and the results are shown in Table 3. Using playing time as a covariate, the worse of 7RST (F = 6.60, p < 0.05, ƞp^2^ = 0.268) were significant and on the other hand when we controlling maturation level standing height (F = 5.2, p < 0.05, ƞp^2^ = 0.2) was significant.

**Table 3:** Results of MANCOVA to control playing time and maturation level of under 15 youth football players.

Variables	Playing time as covariate			Skeletal age as covariate		
	F	P	ƞp^2^	F	P	ƞp^2^
Soccer training (month)	1.1	0.299	0.1	0.6	0.439	< 0.001
Standing height (cm)	2.9	0.102	0.1	5.2	0.035*	0.2
Sitting height (cm)	4.7	0.044*	0.2	1.9	0.190	0.1
Body mass (kg)	2.9	0.104	0.1	< 0.001	0.958	0.0
VO_2max_ (ml kg^-1^ min^-1^)	3.6	0.074	0.1	1.0	0.322	0.1
7RST (s)	1.7	0.206	0.1	0.8	0.376	< 0.001
Best of 7RST (s)	0.3	0.593	< 0.001	3.7	0.071	0.2
Worse of 7RST (s)	6.6	0.019*	0.3	0.5	0.483	< 0.001
Fatigue index (s)	2.8	0.110	0.1	0.5	0.482	< 0.001
CMJ (cm)	1.0	0.329	0.1	0.9	0.347	< 0.001
GH level (ng/dl)	2.5	0.132	0.1	0.1	0.801	< 0.001
IGF-1 level (ng/dl)	1.1	0.308	0.1	1.6	0.215	0.1

*VO*_*2max*_ maximal oxygen uptake, *7RST* 7 repeated sprint test, *CMJ* counter movement jump, *GH* growth hormone, *IGF-1* insulin-like growth factor-1.

*Significant difference at (p ≤ 0.05).

## Discussion

This cross-sectional study aimed to compare the functional capacities and hormonal activities of young soccer players with different maturity and playing time status. The results regarding the morphological and physical performance variables revealed that players advanced in maturation were significantly heavier and had higher values in skeletal age, sitting height, and CMJ than their less mature counterparts. This observation is in line with the findings of previous investigations^7,47–49^. Furthermore, the result indicated that there was no significant difference in playing time status between contrasting maturity groups. The available information on the maturity-related variations in the playing time status of young soccer players is scarce. Nevertheless, in a recent study^50^ similar mean playing times were reported for elite academy players who competed in consecutive age categories. This could be a product of the football culture that the study was conducted in-less based on physicality and more based upon the technical skill level of the players.

The results showed that players, with higher playing time, exhibited a significantly lower fatigue index but higher CMJ values compared to players with less playing time status. Moreover, there were no significant differences in skeletal age and APHV between contrasting playing time groups. Figueiredo et al.^51^ compared the physical, functional, and soccer-specific skills of young elite players in the same team, which was divided into two groups according to their level of seasonal playing time. Their results demonstrated similarities in body size and somatotype between groups. On the other hand, players with more playing time achieved significantly better performance in endurance tests (PACER and 12-min run) and soccer wall pass test. They concluded that besides fitness and sport-specific skill levels, perceived social support may also contribute to higher official playing status. In another comparative study, Sæther and Aspvik ^52^ examined the stress level of young soccer players in different playing time status. Their results indicated that players with lower playing time had greater stress levels when compared to the players with higher playing time.

The results revealed that early-maturing players and players with greater playing time exhibited higher IGF-1 levels than later-maturing players and players with lower playing time. This result is partly in accord with the findings of Hammami et al.^53^ and Nebigh et al.^21^. They reported an increment in IGF-1 level across the pubertal stages in both non-athletic controls and soccer players. It seems that the driving force for higher IGF-1 levels appears to be related to pubertal status rather than load exposure. There is a paucity of knowledge regarding the maturity and playing time status-related differences in hormonal activities of soccer players within the single age category. Besides, other biological factors may also have influences on IGF-1 levels. Therefore, the interpretation of this result should be considered with caution.

There are several limitations to the study. First, it is conducted on a relatively small sample size. Second, other potential technical, tactical, psychological, and sociological determinants that may affect the playing time status of the players are not considered. Third, there is no control group to compare the results. Forth, there is a limitation about the Mirwald et al. equation that this equation predicts maturity offset and age at peak height velocity by subtraction. Nonetheless, these limitations may provide opportunities for future researches. For example, in addition to the functional and hormonal characteristics, variations in soccer skills in male and female players with different playing status and among early, on time, and late-maturing players may yield a better understanding. Despite limitations, this study is one of the few that revealed relationships between maturation status, participation in matches, and fitness status in youth sports players. As practical applications, coaches should consider control maturation status to not wrongly discriminate late mature players, thus improving selection criteria to prevent wrong choices based on current status in long-term player’s development.

## Conclusions

This study is original in the sense that it provides information on the hormonal activities of young elite soccer players. Overall, the results highlighted that early-maturing players and players who were given greater playing time obtained higher values in both CMJ and IGF-1. Furthermore, contrasting playing time and maturity groups did not differ in maturity and playing time status respectively. The findings of the study may provide an insight on the coaches’ evaluation of players and on the possible factors that may affect the future playing status of young male soccer players. Due to their greater body size, muscular strength, and power early maturing players have advantages in team selection. Thus, in order to provide competitive equity and to keep the talented players who are delayed in maturation, physical growth and maturity status should be monitored and taken into account when evaluating the functional capacities.

## Data Availability

The datasets generated during and analyzed during the current study are available from the corresponding author on reasonable request.

## Abbreviations

GH: Growth hormone
IGF-1: Insulin-like growth factor-1
SA-CA: Skeletal age and chronological age
APHV: Age at peak height velocity
CMJ: Countermovement jump
YYIR1: Yo-yo intermittent recovery levels 1
7RST: Seven repeated sprint test
PT1: The first group played less than 50% of the tournament time
PT2: The second group played more than 50% of the tournament time
ML1: Normal maturity
ML2: Early maturity
MANCOVA: Multivariate analyses of covariance
MANOVA: Multivariate analyses of variance
SD: Standard deviation
